# A systematic analysis of evidence for surgically accelerated orthodontics

**DOI:** 10.4317/jced.56048

**Published:** 2019-09-01

**Authors:** Alejandra-Nathaly Mota-Rodríguez, Oralia Olmedo-Hernández, Liliana Argueta-Figueroa

**Affiliations:** 1 Maestría en Ortodoncia, División de Posgrado, Facultad de Odontología, Universidad Autónoma Benito Juárez de Oaxaca, Oaxaca de Juárez, Oaxaca, 65120, México; 2 Cátedras Conacyt - Facultad de Odontología, Universidad Autónoma Benito Juárez de Oaxaca. Av. Universidad S/N, Col. Cinco señores, Oaxaca de Juárez, Oaxaca, 65120, México

## Abstract

**Background:**

Corticotomy is a technique presumed as useful to decrease the time for orthodontic treatment, however, it is necessary to do a systematic review in order to determine if there is enough scientific evidence to back up the use of Corticotomy to accelerate the treatment.

**Material and Methods:**

Data was stockpiled from the electronic database such as PubMed, Cochrane, Scopus y Science Direct, according to the PRISMA linings for systematic reviews, using the following keywords: accelerated movement of the teeth AND osteotomy AND piezocision AND corticotomy AND orthodontics. Only English, Spanish and French language articles that met the criteria needed were included.

**Results:**

In the different accelerated orthodontics techniques, a significant reduction in the total time of orthodontic treatment was obtained. There were no major complications reported in any study. The less invasive procedures had better acceptance.

**Discussion:**

The surgical approaches are not only limited to usual orthodontic treatments, but they have also been used as an alternative for the approach of a palatal fistula in a patient with bilateral cleft lip and palatine orofaciodigital syndrome Type I.

**Conclusions:**

There has been a growing interest in the use of alveolar corticotomies as an adjunct to orthodontic treatment, due to a deeper understanding of its effects and a more robust investigation based on the evidence. All published results indicate a decrease in the total treatment time.

** Key words:**Accelerated movement of the teeth, piezocision, corticotomy AND orthodontics.

## Introduction

Orthodontics is one of the most demanded and prolonged dental treatments. Nowadays there are several techniques to reduce the time of treatment, need for extractions, root resorption and other damage caused to the tissues involved. Therefore, the duration is one of the main reasons why patients leave the treatment before concluding it (1).

Surgery has been intimately involved to include accelerated orthodontics, as a faster means compared to conventional treatment, through the performance of corticotomies, which is defined as a surgical procedure, where the cortical bone adjacent to the alveolar processes is cut, pierce or alter mechanically, this procedure induces a decrease in bone mineral density which is known as osteopenia. In 1959, Kole introduced this technique as a way for rapid movement of the teeth, however, due to how traumatic was this method, Duker (1975) used Kole´s basic technique in beagle dog making some modifications; subsequently, Suya (1991) reported on the orthodontic treatment assisted by corticotomy performed in 395 Japanese adult patients. Suya´s technique differed from Kole´s by the substitution of horizontal cuts corticotomy (2).

Since the introduction of the piezoelectric, that technique has been modified making it less traumatic during the intervention, having the regeneration of hard and soft tissues as an option because it is performed by microincisions and small corticotomies, avoiding full thickness flaps. The technique allows to preserve the soft tissues, thus greatly reducing the possibility of suffering from osteonecrosis, decreasing the healing time, reducing the need for extractions and reducing the treatment time. Therefore, the purpose of this systematic review is to know the advantages and compare the time between conventional orthodontic treatment and accelerated orthodontics by corticotomy.

## Material and Methods

**Figure 1:**
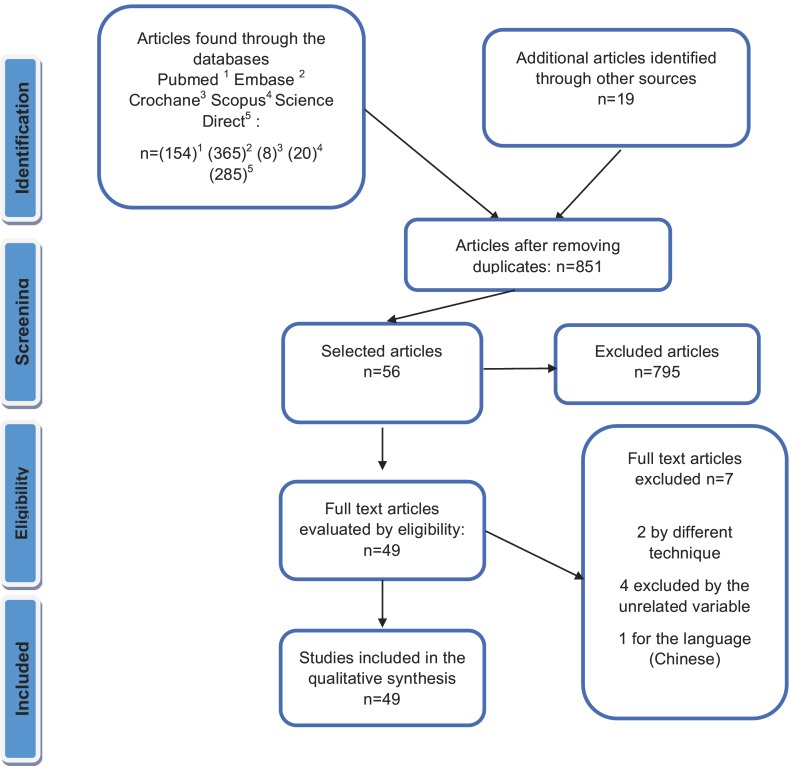
PRISMA flow chart for systematic review.

In the present systematic review, the available data on accelerated orthodontics were compiled from the electronic database PubMed, Cochrane, Scopus, Science Direct, according to PRISMA statement for systematic reviews (Fig. 1), the search for accelerated orthodontics by corticotomy was performed during the first months of 2019. Two reviewers searched and extracted the data independently, previously standardized according to PRISMA and checklist was drawn up to evaluate the studies, which was compared to the end of the search. The electronic search was performed with the following keywords: accelerated movement of the teeth AND piezocision AND corticotomy AND orthodontics. The eligibility of the studies that could be included in the review was determined by reading the title and the summary of each article identified in the search, and then the complete text of the selected articles was retrieved. To limit the articles susceptible of being revised in depth, the following inclusion criteria were taken: Full-text articles in English, French, and Spanish focused on the objective of this review, articles published in indexed journals, articles without restriction on the age of publication, original articles with design of randomized controlled trials, controlled clinical trials and case series on orthodontics facilitated by corticotomy in healthy patients. The evaluation of the quality of the articles was determined considering the design of the original and methodologically coherent research and published in journals indexed in Journal Citation Reports (JCR). The bibliographic references of the articles consulted were also considered as manual search, as relevant articles not included in the electronic search if they had the inclusion criteria of the search. The extraction, collection, management, and analysis of data consisted in the description of the relevant evidence which is presented in the flow chart according to PRISMA. In addition, a critical evaluation of the results is presented in an orderly manner under appropriate headings for each one of them.

## Results

Taking into account the 49 selected articles, 100% are in journals indexed in JCR, 82% are classified in quartiles 1 and 2, so it is assumed that the quality of the articles reviewed is at least acceptable.

After making the selection criteria, a total of articles n=31 (100%) with a total of 618 participants was obtained, with an age ranging from 11 to 46 years, with an average of 24 years. Of these studies, 14 (45.1%) reported the technique of accelerated orthodontics with piezoelectric, 8 (25.8%) were performed with conventional corticotomy, 2 (6.4%) with orthognathic surgery, 2 (6.4%) micro perforations, 2 (6.4%) osteotomy, 1 (3.2%) alveolar distraction, 1 (3.2%) interseptal reduction and 1 (3.2%) accelerated osteogenic orthodontics.

**Figure 2:**
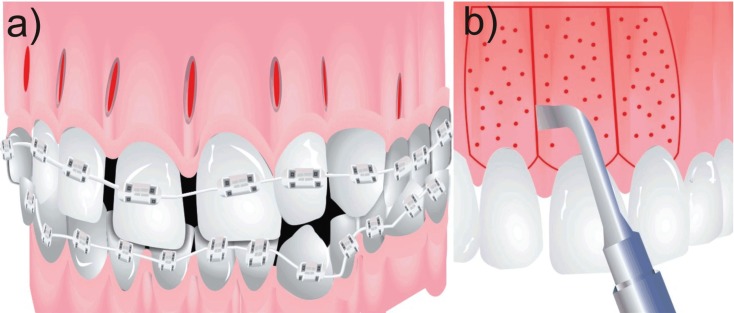
Systematic representation of: a) micro osteo perforations and b) corticotomy.

In the group of conventional corticotomies, in 90% of the cases, a full-thickness flap was used, this technique was used to treat mainly class II and III malocclusions; and in a lesser proportion, retained canines and maxillary protrusion, only one case presented loss of anchorage and the other studies did not present major complications. The treatment reduction time was significant as shown in  Table 1,  Table 1 continue,  Table 1 continue-1. The second group evaluated were the procedures that were performed with piezoelectric, in which the approach of the flap decreased, the treated conditions share a degree of importance between class II and III and cases treated with moderate to severe crowding, the degree of acceptance was satisfactory and the degree of discomfort decreased because it is a less invasive procedure, as in the conventional corticotomy group the treatment reduction time is satisfactory and significant compared to the conventional orthodontic technique as is shown in Figure 2.

**Table 1:**
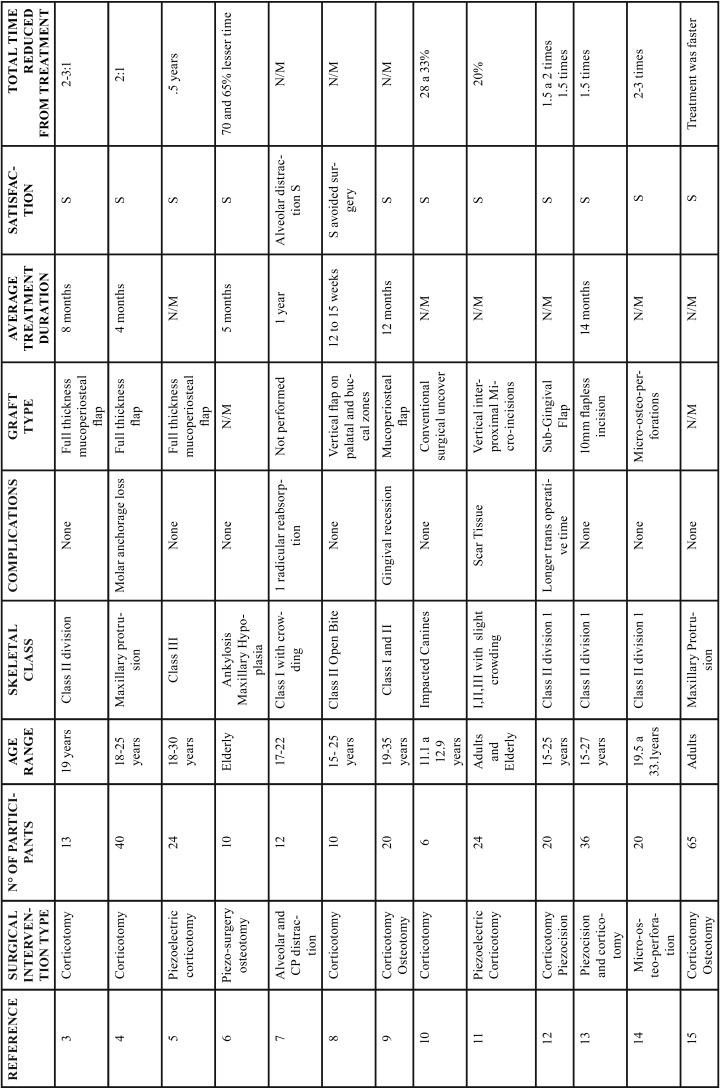
Results of studies performed with corticotomies.

**Table 1 continue:**
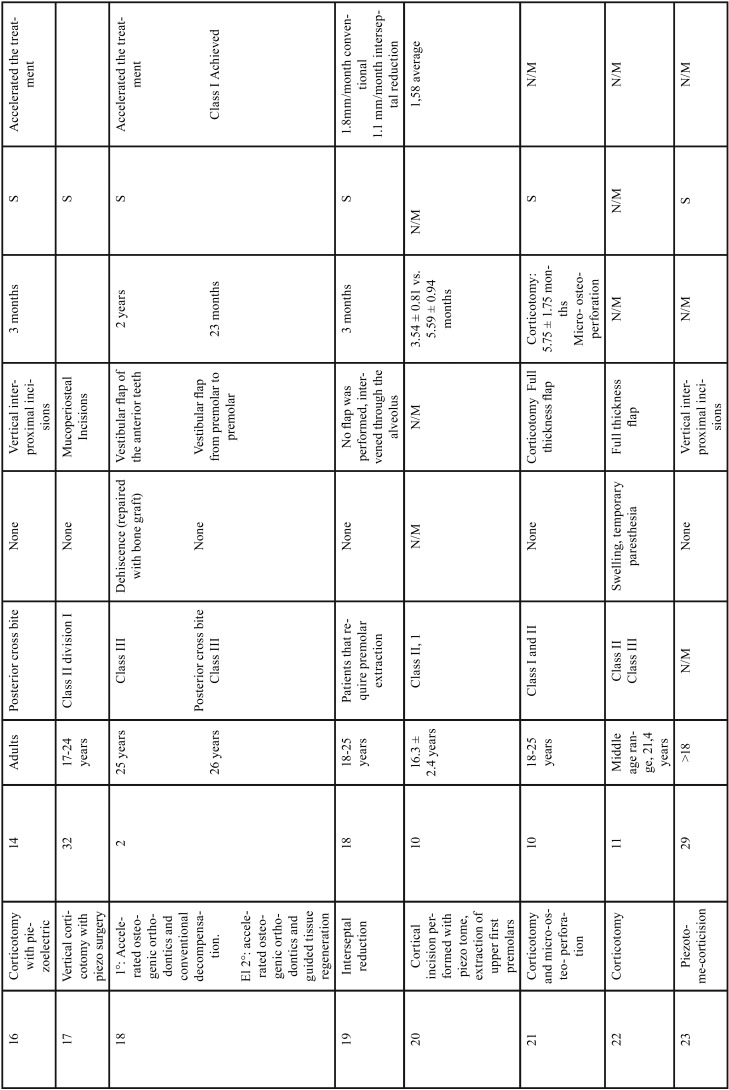
Results of studies performed with corticotomies.

**Table 1 continue-1:**
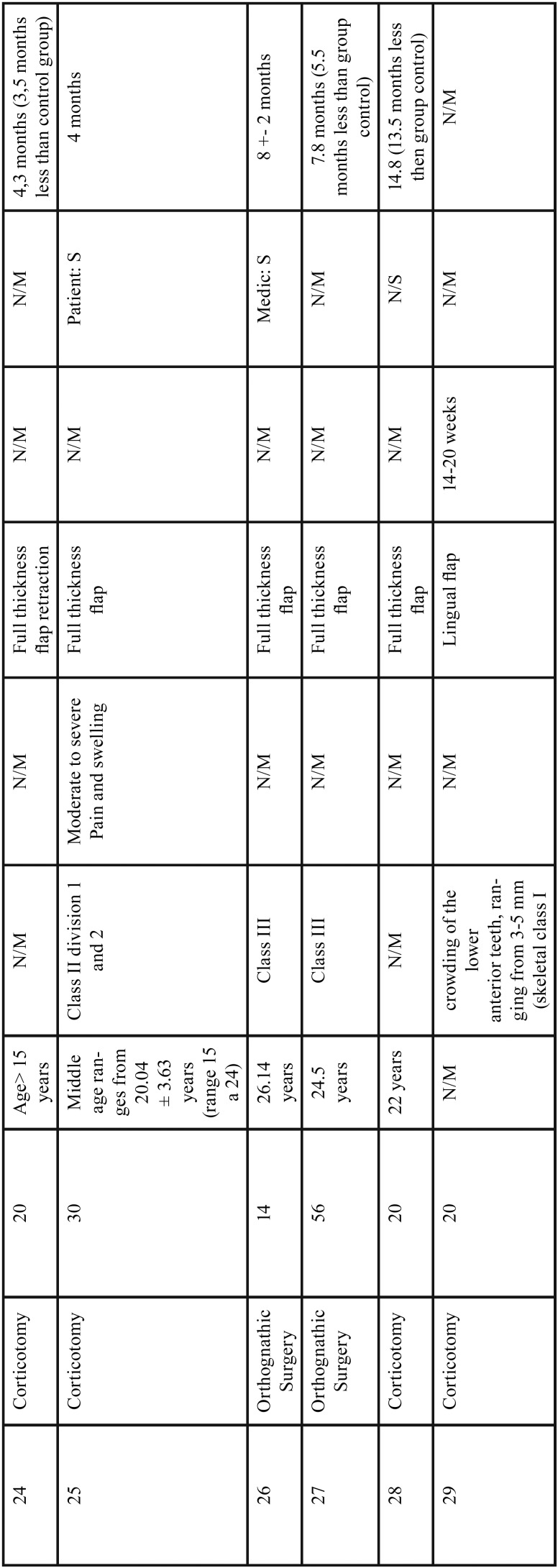
Results of studies performed with corticotomies.

The corticotomies that took place at the same time as the orthognathic surgeries aimed to treat patients with skeletal class III, using highly invasive flap procedures, since this is required by the procedure, resulting in a significant reduction in treatment time compared to patients of the control groups.

The review of original research about corticotomies through microperforations was also included in this study, these treated groups were those that received less trauma at the time of performing the procedure, since the technique has been modified until it becomes part of minimally invasive dentistry and not compromising the oral tissues in a relevant way.

## Discussion

Due to the diversity of the variables and the disparities in the procedures of the articles selected for the present review, it was not possible to perform a meta-analysis of the data.

Currently, it is very important for both patients and clinicians, to reduce the time that an orthodontic treatment is performed, and in many cases, patients are not willing to undergo orthognathic surgeries because of the risks and costs involved, so they look for other options to avoid them as much as possible. Several procedures have been developed and used, the techniques most employed are those that decrease bone density, because orthodontic appliances allow the teeth to move more, when the bone’s resistance to the dental roots is lower.

In the orthodontic area, there are many obstacles to reach the desired results such as retained canines, dental ankylosis, dental discrepancies, maxillary bi-protrusions, extreme skeletal classes, just to mention a few, however, at the same time, there are an increment on research to resolve this type of problems, diverse protocols have been developed from these points and there are many studies that focus on the orthodontic movements facilitated surgically: corticotomy, osteotomy and micro-perforations ( Table 2).

**Table 2:**
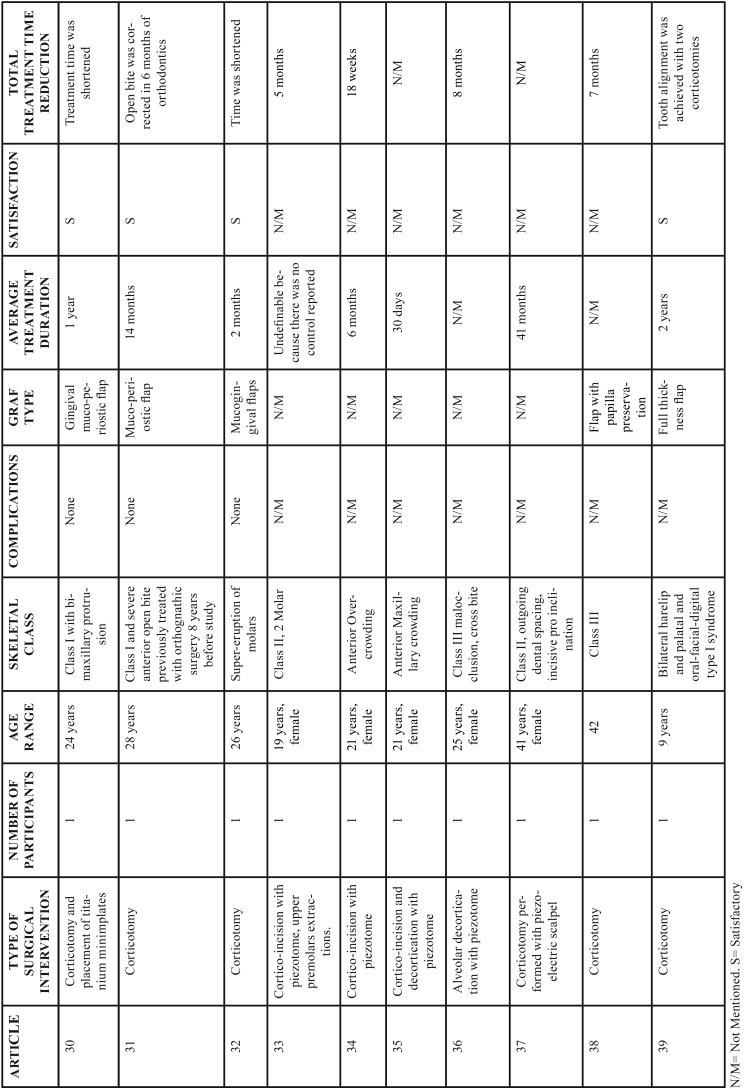
Infrequent applications of corticotomy.

The corticotomy has also evolved and some studies have shown the results of this approach using various techniques, most of them need full mucoperiosteal flaps, giving excellent results. Wu J, *et al.* (5) reported that, in patients with class III, orthodontic time can be reduced up to six months. Sakthi, *et al.* (40) evidence that, when performing premolar extractions and immediately corticotomies in the alveolus, the orthodontic treatment time can be reduced by up to 50%.

Undoubtedly one of the reasons why an orthodontic treatment is delayed, is the presence of dental ankylosis. For a long time, this was a limitation, however, Bertossi (6) reports the realization of osteotomic lines laterally and apically to the root dental in the bone of ankylosed dental organs, and their results indicate reduction of the treatment time having very low incidence of side effects.

On the other hand Abbas (12) and Alfawal (13), carried out studies on class II malocclusions division I. Abbas, indicates that corticotomies in treatments whose biomechanics includes extraction of the maxillary first premolar and subsequent rapid canine retraction, are auxiliary to effective treatments that reduce the time required for canine retraction and decrease the resorption of roots in adults. The orthodontics facilitated by the corticotomy is 1.5 to 2 times faster than conventional orthodontics, while with piezocision it was 1.5 times faster than conventional orthodontics. Alfawal, (13) reported the treatment in 36 patients who required extraction of the first upper premolars, followed by canine retraction with piezocision and corticotomy without laser-assisted flap, the results showed that the treatment method is effective to accelerate canine retraction without significant unfavorable effect on anchoring or canine rotation during rapid retraction.

The intrusion of molars can become very difficult in certain cases, so it is necessary to use other resources for acceleration, since movements with orthodontic appliances are not enough. Moon (34), made a clinical case of a patient with extruded molars, intruded 3.0 mm the first molar and the second molar 3.5 mm in only 2 months of treatment, keeping them for 11 months under observation without suffering recurrence.

Surgical approaches are not only limited to usual orthodontic treatments, Yen S (39), gives us a very peculiar case, it addressed a palatal fistula in a patient with bilateral cleft lip and palatine oral-facial-digital syndrome type I, after bone transport and a series of corticotomy lines, it was achieved, that the sites of the cleft were grafted, the palatal fistula was faced, the lateral segments were expanded and the alignment of the teeth was improved. There is certainly much to investigate about the use of surgical approaches in this type of unconventional cases.

It is recommended the use of corticotomies in patients with orthodontic appliances with short roots, to avoid reabsorption caused by bone resistance during dental movements, patients with loss of bone tissue or hypoplasia of the jaws that require bone remodeling, patients with dental ankylosis for decrease the alignment time, patients with teeth retained to accelerate their anatomical positioning within the arches, patients with extreme skeletal classes to avoid orthognathic surgery and patients with severe crowding.

All the published results seem to go in the direction of the possibility of diminishing the total time of treatment. In the context of adequate patient selection, corticotomies can be a powerful and safe tool to improve the quality and duration of orthodontic treatments.

## Conclusions

There has been a growing interest in the use of alveolar corticotomies as an adjunct to orthodontic treatment, due to a deeper understanding of its effects and a more robust investigation based on the evidence. The biological stimulus produced by corticotomies is reflected in the trabecular bone and therefore provides an opportunity to accelerate certain orthodontic movements. The different techniques of accelerated orthodontics reduce the phase of hyalinization that delays dental movement, which has led to a better acceptance of the patient and the clinician.
